# Assessment of p53 in Endometrial Carcinoma Biopsy and Corresponding Hysterectomy Cases in a Real-World Setting: Which Cases Need Molecular Work-Up?

**DOI:** 10.3390/cancers17091506

**Published:** 2025-04-29

**Authors:** Marie-Lisa Eich, Janna Siemanowsk-Hrach, Uta Drebber, Nicolaus Friedrichs, Peter Mallmann, Christian Domröse, Dominik Ratiu, Sabine Merkelbach-Bruse, Reinhard Büttner, Alexander Quaas, Birgid Schömig-Markiefka

**Affiliations:** 1 Institute of Pathology, University Hospital of Cologne, University Cologne, Faculty of Medicine, 50937 Cologne, Germany; 2 Institute of Pathology, Charité-Universitätsmedizin Berlin, Corporate Member of Freie Universität Berlin and Humboldt-Universität zu Berlin, 10117 Berlin, Germany; 3 Department of Obstetrics and Gynecology, University Hospital Cologne and Medical Faculty, 50937 Cologne, Germany

**Keywords:** p53, endometrial carcinoma, biopsy, hysterectomy

## Abstract

**Background/Objectives**: The WHO classifies endometrial cancer into molecular subgroups, including tumors with abnormal p53 expression. In clinical practice, the p53 status can be assessed using either biopsy or hysterectomy samples. This study aimed to evaluate the correlation between p53 immunohistochemistry (IHC) and *TP53* molecular status in both tissue types and assess interobserver variability among five pathologists. **Methods**: We analyzed 98 endometrial biopsies and 86 hysterectomy samples from 101 patients (83 matched cases). Five pathologists with varying experience independently assessed p53 IHC staining. Targeted panel sequencing was performed on 169 of 184 cases. **Results**: Pathologists recommended molecular *TP53* analysis in 14.3–23% of biopsies and 3.5–32.6% of hysterectomy cases. For biopsy samples, kappa values for IHC-molecular agreement ranged from 0.61 to 0.94, while hysterectomy cases showed higher agreement (kappa 0.83–1.0). When all five pathologists agreed on the IHC p53 status, molecular analysis confirmed the same results in all evaluable cases (no mutation/wild-type: 42/42 biopsy, 42/42 hysterectomy; mutated: 3/3 biopsy, 5/5 hysterectomy). **Conclusions**: Despite variability in staining intensity between tissue types, p53 IHC showed complete agreement with *TP53* molecular status when all pathologists concurred. Experienced pathologists demonstrated higher concordance with molecular results, though molecular testing was necessary in up to one-third of cases. Adequate training can improve IHC reliability, but molecular confirmation is essential for equivocal cases.

## Simple Summary

Endometrial cancer can be classified based on molecular markers, including the tumor suppressor p53 protein, which plays a key role in regulating cell division and predicting patient outcomes. This study evaluated how well p53 immunohistochemistry (IHC), a common lab test, reflects the actual *TP53* gene status and whether the test results are interpreted consistently across different pathologists. Five pathologists analyzed 2 types of tissue samples—98 biopsy and 86 hysterectomy samples—with genetic testing performed in all cases. When all five experts agreed on the IHC results, they matched 100% with genetic testing. However, in up to one-third of cases, molecular testing was needed for a clearer answer. Accuracy was higher among experienced pathologists. The findings indicate that p53 IHC, with proper training, can be a reliable method for assessing the status of the p53 tumor suppressor. Still, molecular testing remains essential when IHC results are unclear to ensure accurate diagnosis and guide appropriate treatment decisions.

## 1. Introduction

Over 420,242 women were diagnosed with uterine cancer worldwide in 2022 [1], with endometrial carcinoma being the most frequent cancer type comprising over 90% of cases [2,3]. In the localized stages (FIGO stage I and stage II), survival rates are generally good, reaching 95% [4].

Histologically, endometrial carcinoma subtypes include endometrioid, serous, clear cell, undifferentiated, mixed cell, mesonephric, squamous cell, mucinous, mesonephric-like, and carcinosarcoma. Endometrioid adenocarcinoma is, with about 85% of cases, the most common subtype, followed by serous and clear cell carcinoma [5]. Based on the molecular and prognostic classification of endometrial cancer by The Cancer Genome Atlas [6], the WHO has incorporated molecular features into its treatment guidelines [3,7]. A key component of stratification into molecular risk groups is the evaluation of *TP53* mutations. Patients with *TP53*-altered/p53 abnormal tumors have the worst outcome among endometrial carcinoma patients, causing 50 to 70% of endometrial carcinoma-related deaths [8,9,10,11]. Previous studies have shown that p53 immunohistochemistry (IHC) can serve as a surrogate marker for an underlying *TP53* mutation in endometrial cancer samples [12,13,14]. The Proactive Molecular Risk Classifier for Endometrial Cancer (ProMisE) incorporates p53 expression as a surrogate for *TP53* mutations, representing the copy-number high group [12,15,16,17]. However, other studies have shown discrepant results between the molecular *TP53* status and p53 protein expression in 5% to 25% of cases [13,14,18]. Currently, abnormal p53 includes overexpression, the so-called null pattern [14,19], and cytoplasmic staining [20], but only the first two patterns are adopted in the European Society of Gynecological Oncology (ESGO), the European Society for Radiotherapy and Oncology (ESTRO), and the European Society of Pathology (ESP) recommendations [21,22]. The cytoplasmic staining pattern in endometrial carcinoma is considered a mutant pattern when more than 80% of the tumor exhibits such staining. This pattern is caused by mutations in the C-terminal domain of *TP53*; however, it occurs in only approximately 2% of endometrial carcinoma cases [23].

In the aforementioned studies, correlation analysis between p53 IHC and *TP53* mutational status were conducted in a research setting, with a focus on biopsy-only or hysterectomy-only samples evaluated by specially trained gynecologic pathologists. However, in a real-life clinical setting, only biopsy or hysterectomy tissue is sometimes available for further analysis. Furthermore, IHC scoring in the clinical setting is not always performed by a subspecialized gynecologic pathologist.

In contrast with previous studies, we investigated p53 immunohistochemistry in biopsy and corresponding hysterectomy tissues in a real-life setting to determine whether both tissues are equally suitable for routine clinical workflows. Additionally, we examined the interobserver variability among pathologists with different levels of experience in a real-life clinical practice setting and the correlation between p53 immunohistochemistry and molecular pathological *TP53* mutation analysis to identify cases where immunohistochemical p53 status assessment alone is insufficient.

## 2. Materials and Methods

### 2.1. Patient Cohort

This study was approved by the institutional review board of the University Hospital Cologne (number 22-1364). Written informed consent was obtained from all patients prior to their inclusion in this study.

Surgical pathological archives were searched between 2019 and 2022 for endometrial carcinoma cases, with an emphasis on cases in which both biopsy and hysterectomy samples were available. Ninety-eight endometrial biopsy samples and eighty-six hysterectomy samples were selected for analysis. For 83 patients, both biopsy and hysterectomy samples were available for further study.

### 2.2. Immunohistochemical Assessment

Whole-slide biopsy and hysterectomy samples were subjected to immunohistochemistry for p53 (clone: DO-7, dilution: 1:1800, manufacturer: Agilent Dako, Santa Clara, CA, USA, catalogue number GA616) following the manufacturer’s protocol. Immunolabeling was detected via anti-mouse or anti-rabbit horseradish peroxidase-conjugated secondary antibodies, and the staining was developed via 3,3′-diaminobenzidine. Tonsil tissue served as a negative control. Representative images are provided in Supplementary Figure S1.

P53 was considered mutant if no staining was visible, strong nuclear expression was detected in more than 80% of the nuclei, or strong cytoplasmic staining was present. A varying staining pattern across tumor nuclei was considered a wild-type staining pattern [6].

Five pathologists independently evaluated p53 staining in biopsy and resection samples from patients with endometrial carcinoma. Three pathologists (pathologists 1 to 3, B.S-M., A.Q., U.D) have over fifteen years of experience working at a tertiary care center in Germany. One pathologist has over fifteen years of experience working in both a tertiary care center and private practice (pathologist 4, N.F.). Pathologists 1 and 2 are experienced in gynecologic pathology. The fifth pathologist is a resident in training with four years of experience at a tertiary care center (pathologist 5, M-L.E).

### 2.3. Parallel Sequencing (Next-Generation Sequencing, NGS)

Prior to DNA extraction, an experienced pathologist estimated the tumor cell content via hematoxylin–eosin (H&E)-stained slides. The corresponding unstained tumor areas were macrodissected from 10 µm thick formalin-fixed paraffin-embedded (FFPE) tissue sections. After overnight proteinase K digestion, the DNA was isolated with the Maxwell^®^ RSC FFPE Plus DNA Kit (Promega, Mannheim, Germany) on the Maxwell^®^ 48 (Promega) following the manufacturer’s instructions [24]. For hybrid capture-based next-generation sequencing (NGS), the DNA concentration was measured with a Qubit 2.0 fluorometer (Thermo Fisher Scientific, Waltham, MA, USA) via the Qubit dsDNA HS Assay Kit. After enzymatic fragmentation, library preparation and target enrichment were performed via a customized hybrid capture-based Twist Bioscience Panel including *TP53* Exons 2-11 following the manufacturer’s instructions (Twist Bioscience, South San Francisco, CA, USA).

Libraries were sequenced on a NextSeq instrument (Illumina, San Diego, CA, USA) with a NextSeq Reagent Kit V2 (300 cycles) (Illumina) according to the manufacturer’s recommendations.

### 2.4. Data/Statistical Analysis

The data were analyzed with R 4.1.2 (The R Foundation for Statistical Computing, Vienna, Austria). Cohen’s kappa was calculated via the DescTools package in R. Fleiss’ kappa was calculated via the irr package in R. Alignment and variant calling of the NGS data were performed via an in-house pipeline. BAM files were visualized via the Integrative Genomics Viewer (http://www.broadinstitute.org/igv/ (Version, 2.5.5 (6) 14 May 2013, accessed on 1 December 2022) Cambridge, MA, USA). For variant detection, a cutoff of 5% allelic fraction with a minimum coverage of 200 was used.

## 3. Results

### 3.1. Clinicopathological Features

**Figure 1:**
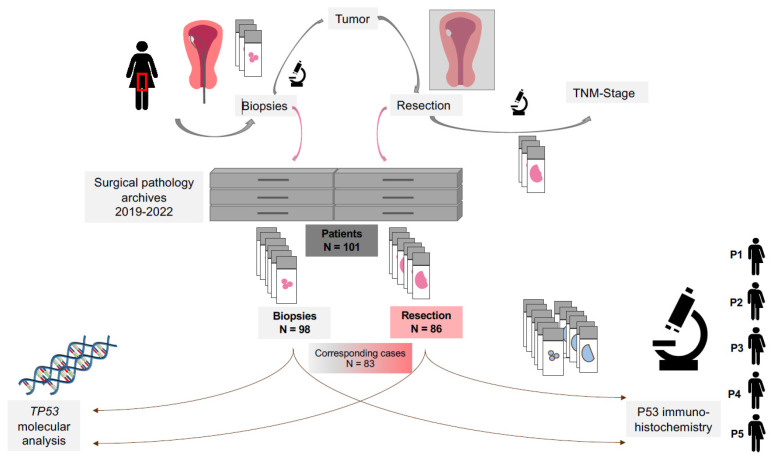
Workflow for p53 and *TP53* analysis in patients with endometrial carcinoma. Patients with endometrial cancer first undergo an endometrial biopsy for diagnostic purposes. The biopsy samples are evaluated and stored in the surgical pathology department. Following diagnosis, patients typically undergo a hysterectomy as part of their treatment. The final tumor stage and subtype are then determined in the pathology department. For the present study, surgical pathology archives were reviewed for endometrial cancer samples. A total of 184 cases from 101 patients were retrieved, including 98 biopsy and 86 resection samples. For 83 patients, both biopsy and resection samples were available. These samples were subjected to p53 immunohistochemistry and *TP53* molecular analysis. Abbreviations: P1—pathologist 1; P2—pathologist 2; P3—pathologist 3; P4—pathologist 4; P5—pathologist 5. TNM-Stage—tumor (T), nodal (N), metastasis (M)-stage.

We included 101 patients with endometrial cancer in this study, with 98 biopsy samples and 86 resection samples (clinicopathological parameters are shown in Table 1). For 83 patients, both biopsy and corresponding hysterectomy samples were available for further analysis (see Figure 1). The majority of patients had an endometrioid carcinoma subtype (95.1% of biopsy and 92.0% of resection cases). Tumors from 29 patients were mismatch repair deficient (28.7% dMMR/MSI; see Table 1).

### 3.2. Immunohistochemical Assessment of p53 Status in Biopsy and Resection Samples in Correlation with Molecular TP53 Status in Patients with Endometrial Carcinoma

**Figure 2:**
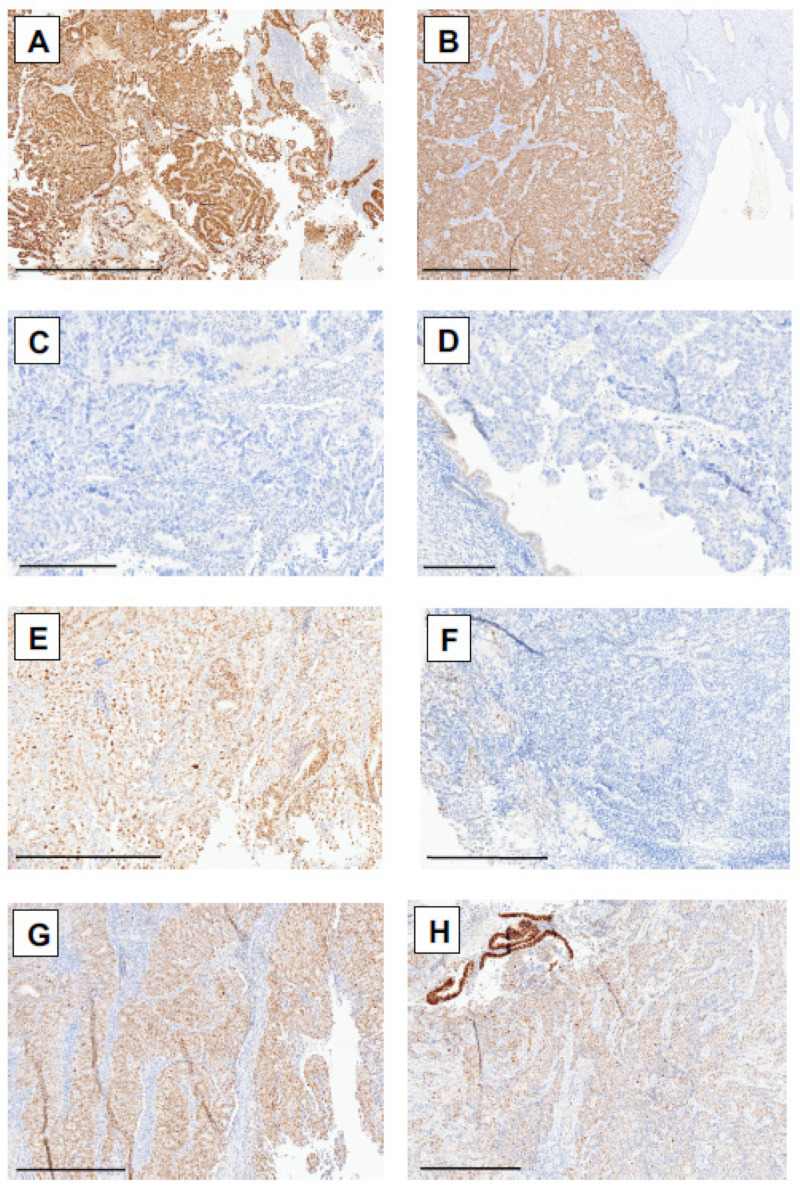
p53 immunohistochemical staining patterns in patients with endometrial cancer in corresponding biopsy and hysterectomy samples. (**A**) Overexpression of p53 in patient 8 in biopsy and (**B**) hysterectomy samples. (**C**) Null p53 staining pattern in biopsy and (**D**) hysterectomy samples from patient 17. (**E**) Wild-type staining pattern in biopsy and (**F**) hysterectomy samples from patient 96. (**G**) Wild-type staining pattern in patient 53 in the biopsy specimen and (**H**) focal overexpression area in the hysterectomy specimen, showing *TP53* mutation via molecular analysis. Scale bars: (**A**,**B**): 1 mm; (**C**,**D**): 300 µm; (**E**–**H**): 500 µm.

**Figure 3:**
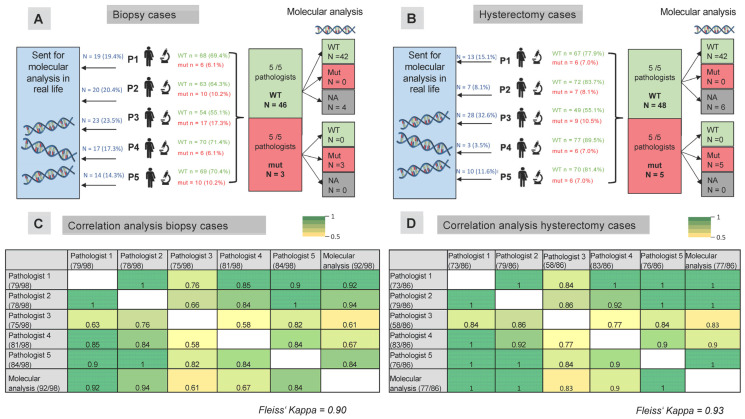
Correlation analysis of p53 immunohistochemical analysis and molecular *TP53* status in biopsy and hysterectomy samples from patients with endometrial carcinoma. Five pathologists independently evaluated p53 staining in biopsy (**A**) and hysterectomy (**B**) samples from patients with endometrial carcinoma. In biopsy cases (**A**), 14.3% to 23.5% of cases were sent for molecular analysis in a real-life setting, where pathologists did not rely solely on immunohistochemical analysis to define the p53 status. In hysterectomy cases (**B**), molecular analysis would have been performed in 8.1% to 32.6% of cases. When all five pathologists agreed on the p53 status, the results were concordant with the results of the molecular analysis. Correlation analysis of the p53 status determined by pathologists via immunohistochemistry is shown for biopsy samples (**C**) and hysterectomy samples (**D**). Fleiss’ kappa was 0.90 for the biopsy samples and 0.93 for the hysterectomy samples. The numbers in the table represent individual Cohen’s kappa values. The numbers in brackets indicate the number of cases in which each pathologist determined the p53 status. For example, pathologist 1 determined the p53 status in 73 of 86 biopsy samples, while the remaining 13 samples were sent for molecular analysis. Abbreviations: WT—wild-type, mut—mutated.

Five pathologists independently evaluated p53 staining in biopsy and resection samples from patients with endometrial carcinoma (the results for each patient can be found in Supplementary Tables S1 and S2). The staining intensities varied between the biopsy and hysterectomy samples (representative cases are shown in Figure 2), with generally weaker staining in the hysterectomy samples due to fixation artifacts. Owing to ambiguous staining patterns, pathologists require molecular analysis for the *TP53* status in 14.3% to 23% of biopsy cases and 3.5% to 32.6% of resection cases (see Figure 3A). For the purposes of this study, all cases underwent molecular analysis, and the results were available for 92 biopsy samples (93.9%) and 77 resection samples (89.5%).

Except for pathologist 3, who required a greater number of molecular analyses in biopsy cases than in resection cases (see Figure 3), the other pathologists required a similar proportion of molecular analyses in both biopsy and hysterectomy cases. Pathologist 5 (a resident in training) performed molecular analysis on a similar number of patients as the experienced tertiary center pathologists.

Overall, 14 biopsy and 8 resection samples presented mutations in the *TP53* gene (the corresponding IHC staining patterns are shown in Supplementary Table S3). In four patients, matching *TP53* mutations were detected in both the biopsy and the corresponding hysterectomy samples. In two hysterectomy cases (R85 and R88; see Supplementary Table S3), the same *TP53* mutation as found in the biopsy was present at an allelic fraction of 1%. Due to this low allelic frequency, these cases were classified as wild-type. In four additional cases, a *TP53* mutation was identified only in the biopsy sample. Two of these were mismatch repair-deficient based on immunohistochemical analysis (MSI). In the biopsy sample, p53 IHC also showed only focal overexpression. One case showed a wild-type p53 immunohistochemical staining pattern, but molecular analysis revealed a splice site variant of unknown significance.

Compared with the molecular *TP53* status, individual kappa values ranged considerably from 0.61 to 0.94 for biopsy samples, considering only those where pathologists relied on immunohistochemical evaluation without performing molecular analysis (see Figure 3C). For resection cases, the kappa values ranged from 0.83 to 1.0 (see Figure 3D). Pathologists 1 and 2, who had the most experience in p53 evaluation, showed the highest agreement with the molecular results. Overall, taking all pathologists’ and molecular testing results into account, a Fleiss’ kappa of 0.90 was observed for patients who underwent a biopsy, and 0.93 was observed for patients who underwent a hysterectomy.

All five pathologists agreed on p53 status in 49/98 biopsy cases and 53/86 hysterectomy cases (see Supplementary Tables S1 and S2). In cases where all five pathologists scored a case as wild-type in biopsy samples (n = 46), molecular analysis confirmed wild-type status in all cases with available molecular results (see Figure 3A). The three cases that were scored as mutated by all five pathologists were indeed mutated based on molecular analysis. Similarly, for the resection samples, all samples that were scored as wild-type via immunohistochemistry by all five pathologists were confirmed as wild-type via the available molecular analysis. Similarly, all patients whose tumors showed a mutational staining pattern for p53 on IHC (null expression/overexpression or cytoplasmic staining), confirmed by all five pathologists, had a single nucleotide variant in the *TP53* gene detected based on next generation sequencing (see Figure 3B).

## 4. Discussion

The current study was carried out in a tertiary care center with access to molecular diagnostics. Five pathologists with varying levels of experience and exposure to gynecologic malignancies, ranging from residents in training (4th year) to those with more than 20 years of practice at a university hospital, independently evaluated p53 IHC in 98 endometrial cancer biopsy samples and 86 endometrial cancer hysterectomy samples. Without any special training, pathologists ordered molecular testing in up to one-third of cases (ranging from 3.5% to 32% of cases), with a surprisingly higher frequency in biopsy samples than in hysterectomy samples. Prior to the present study, we expected a greater frequency of the need for molecular testing in hysterectomy cases because of more fixation artifacts and weaker p53 staining in general.

For study purposes, all specimens were molecularly tested for *TP53* mutations. All five pathologists agreed on the p53 status in 50% of the biopsy samples and 62% of the hysterectomy samples. For these patients, the p53 IHC status was 100% concordant with the molecular results. The highest agreement with molecular analysis was achieved by the pathologists with the most experience in p53 evaluation in gynecologic malignancies (kappa values for biopsy samples compared with molecular *TP53* status: 0.92 and 0.94; kappa values for hysterectomy samples: 1.0 and 1.0). However, p53 scoring by the residents in training yielded equal results for hysterectomy samples, with a kappa value of 1.0 compared with the molecular *TP53* status, whereas the kappa value for biopsy samples was 0.84. While the residents in training focused on immunohistochemical evaluation approaches during their training, the remaining two pathologists were general pathologists without a special focus on IHC scoring. Therefore, their agreement with p53 scoring compared with the *TP53* molecular status was 0.61 and 0.67 in biopsy samples.

P53 immunohistochemistry (IHC) can serve as a surrogate parameter for an underlying *TP53* mutation in endometrial cancer samples [12,13,14]. However, discrepant results between the molecular *TP53* status and p53 protein expression can occur in 5% to 25% of cases [13,14,18]. In a clinical trial setting, the agreement between p53 IHC and *TP53* molecular analysis was only 90.7% [13]. Hoang et al. reported in their agreement analysis of ProMisE classification among seven pathologists that concordant results for the p53-abnormal group were observed in only 40% of cases [25].

In a meta-analysis of five studies, Raffone et al. reported that p53 overexpression demonstrated a pooled sensitivity of 0.79 as a surrogate for *TP53* mutations by NGS and a pooled specificity of 0.96 [16]. However, the cutoff criteria for abnormal p53 IHC results vary among studies [16,19]. Most recent studies have used a strong staining threshold of 80% or greater in tumor cell nuclei to define p53 overexpression [12,18,19]. Talhouk et al. investigated the concordance of ProMisE classes between biopsy and hysterectomy samples in a cohort of 57 corresponding cases. They reported a high concordance of classes between biopsy and hysterectomy samples. Consequently, a high concordance of p53 IHC between biopsy and hysterectomy samples was also observed. Given the nature of the ProMisE classifier, no molecular *TP53* testing was conducted in their study [26]. Similarly, Stello et al. found a high concordance of p53 staining between biopsy and hysterectomy specimens in their study of 48 patients with endometrial carcinoma [27].

A known limitation of p53 IHC staining is the variation in staining intensity between different laboratories. A study by Plotkin et al. revealed that weak staining patterns, as well as wild-type and loss of expression, can cause confusion between wild-type and overexpression strains. Therefore, even an experienced gynecologic pathologist may classify the same case differently when it is stained in a different laboratory [28]. Koebel et al. highlighted the importance of p53 antibody calibration and the establishment of a standardized protocol for p53 staining in each laboratory. They also noted that weaker staining protocols might lead to overcalling cases as “wild-type” that actually show an “overexpression” pattern, or misclassifying cases as “mutant with null pattern” when they are in fact wild-type cases [19].

In contrast to our study, all of the aforementioned studies were conducted in a research setting by specially trained gynecologic pathologists. As a result, these studies were optimized for the highest agreement between p53 IHC and *TP53* mutational status. However, in a real-world clinical workflow, diagnosis and p53 status are often determined by general pathologists. In our study, we show that pathologists, despite having years of clinical experience, only had substantial agreement in p53 IHC scoring for endometrial carcinoma biopsy specimens with molecular analysis (kappa values < 0.80) if no special training was received. Furthermore, the assessment of p53 status and histological subtype and grade is usually recommended for biopsy samples [21]. In our study, a *TP53* mutation was observed in four cases exclusively in the biopsy samples, while the corresponding hysterectomy specimens lacked the mutation. Additionally, two cases showed the *TP53* mutation at a low allelic frequency, supporting the rationale for testing the biopsy. Furthermore, two cases showed the *TP53* mutation only at a low frequency, giving the rationale for testing the biopsy. However, we demonstrated in our study that the p53 IHC status correlated more reliably with the *TP53* molecular status in hysterectomy samples, suggesting that re-evaluating the p53 status in the final hysterectomy sample may be useful if the initial biopsy is scored as wild-type.

The current study was conducted in a real-world setting and, to the best of our knowledge, is the only study investigating the interrater agreement of p53 IHC status among pathologists with different levels of experience. This is also one of the largest studies to allow comparative p53/*TP53* analysis between biopsy and hysterectomy samples in endometrial carcinoma. However, this study is not without limitations. Since the primary focus was on the real-world correlation of p53 IHC and *TP53* mutations, this study lacked outcome analysis. Although all contributing pathologists have different levels of work experience in both private practice and academic settings, laboratory testing was performed in a single center, thus not accounting for the variability in p53 staining protocols across different institutions. Pathologists in tertiary care centers may encounter referral cases with varying staining protocols. As endometrioid endometrial carcinoma accounts for over 85% of all endometrial cancers [3,5,29], this study focused on this most common subtype, and included only five serous carcinomas. Given that cases were selected from routine clinical workflows, most endometrioid endometrial carcinoma cases were of low-to-intermediate grade, consistent with findings in the literature [5,30]. As a result, only 14 biopsy samples and 8 hysterectomy samples presented abnormal p53 staining. Furthermore, for each tumor specimen, only one slide was analyzed. Due to intratumoral heterogeneity, potential subclonal mutations or aberrant staining patterns might not have been captured.

Molecular testing is not available in all laboratories or institutions and is significantly costlier than IHC analysis. Owing to the high agreement between the p53 IHC scoring and molecular analysis results of experienced pathologists, p53 can serve as a screening marker for *TP53* mutations in a real-world clinical setting. However, we recommend molecular testing for all cases with any ambiguity. Additionally, pathologists should receive adequate training in p53 IHC scoring for clinical practice. Furthermore, we propose that *TP53* mutational analysis should be performed in equivocal p53 IHC cases. To determine the impact of this approach on patient outcomes, further studies are needed, as our study lacks outcome data.

## 5. Conclusions

International and national guidelines on endometrial carcinoma emphasize the necessity of *TP53*/p53 testing for all patients to better stratify patient risk and inform treatment decisions. In conclusion, *TP53* mutation analysis should be performed in patients with equivocal p53 IHC results. As suggested by Vermij et al., pathologists should receive specialized training for p53 IHC evaluation in patients with endometrial cancer (training tutorial available at http://www.gpec.ubc.ca/p53 (accessed on 28 March 2023)) [13]. Although the staining intensities in our study, and those in previous studies [14], varied between biopsy and hysterectomy samples, both tissue types can be used for determining p53 status, provided that pathologists have high confidence in their p53 IHC results.

## Figures and Tables

**Table 1:** Clinicopathological parameters.

Variables	Values
total number of patients	101
Biopsies Resections	98 86
Age, years Mean (±SD) Min, max	63 (13.5 ± SD) Min. 20 years, max. 90 years
pT stage, resection (n = 86) (%) pT1a pT1b pT2 pT3a pT3b	48 (55.8%) 28 (32.6%) 7 (8.1%) 2 (2.3%) 1 (1.2%)
pN stage, resection (n = 86) (%) pN0 pN1 NA	25 (29.1%) 2 (2.3%) 59 (68.6%)
Tumor size in cm (n = 86) Mean (±SD) NA (n)	3.1 (±2.0) 8 cases
Subtype biopsy (n = 98) (%) Serous Endometrioid	4 (4.1%) 94 (95.1%)
Subtype resection (n = 86) (%) Serous Endometrioid Mixed serous and endometrioid	5 (5.8%) 79 (91.9%) 2 (2.3%)
Grading biopsy (n = 98) (%) 1 2 3	29 (29.6%) 51 (52.0%) 18 (18.4%)
Grading resection (n = 86) (%) 1 2 3	28 (32.6%) 37 (43.0%) 21 (24.4%)
MMR/MSI-status (n = 101) (%) pMMR/MSS dMMR/MSI NA	70 (69.3%) 29 (28.7%) 2 (2.0%)
Molecular results Biopsies Resections	92 (93.9%) 77 (89.5%)

Abbreviation: pT—pathological tumor stage; MSS—microsatellite stable; MSI—microsatellite instable, pMMR—proficient mismatch repair proteins, dMMR—deficient mismatch repair proteins.

## Data Availability

The data supporting the findings of this study are available upon request from the corresponding author. However, due to the sensitive nature of the research, the data are not publicly available, as the participants did not provide written consent for public sharing.

## Supplementary Materials

The following supporting information can be downloaded at: https://www.mdpi.com/article/10.3390/cancers17091506/s1, Figure S1. Negative control for p53 immunohistochemical staining; Table S1. Case wise scoring for p53 in biopsy specimens with endometrial carcinoma; Table S2: Case wise scoring for p53 in hysterectomy specimens with endometrial carcinoma; Table S3. p53 staining pattern in TP53 mutated cases.

## Abbreviations

The following abbreviations are used in this manuscript:

IHC	Immunohistochemistry
dMMR	Deficient mismatch repair proteins
MSI	Microsatellite instable
MSS	Microsatellite stable
pMMR	Proficient mismatch repair proteins
WHO	World Health Organization

## References

[1] Bray F., Laversanne M., Sung H., Ferlay J., Siegel R.L., Soerjomataram I., Jemal A. (2024). Global Cancer Statistics 2022: GLOBOCAN Estimates of Incidence and Mortality Worldwide for 36 Cancers in 185 Countries. CA Cancer J. Clin..

[2] Hosh M., Antar S., Nazzal A., Warda M., Gibreel A., Refky B. (2016). Uterine Sarcoma: Analysis of 13,089 Cases Based on Surveillance, Epidemiology, and End Results Database. Int. J. Gynecol. Cancer.

[3] Matias-Guiu X., Lax S.F., Bosse T., Davidson B., Singh N., Euscher E.D., Raspollini M.R., Liu C., Lortet-Tieulent J. (2020). Endometrioid carcinoma of the uterine corpus. Female Genital Tumors.

[4] Vrede S.W., Kasius J., Bulten J., Teerenstra S., Huvila J., Colas E., Gil-Moreno A., Boll D., Vos M.C., van Altena A.M. (2022). Relevance of Molecular Profiling in Patients with Low-Grade Endometrial Cancer. JAMA Netw. Open.

[5] Espinosa I., D’Angelo E., Prat J. (2024). Endometrial Carcinoma: 10 Years of TCGA (the Cancer Genome Atlas): A Critical Reappraisal with Comments on FIGO 2023 Staging. Gynecol. Oncol..

[6] Kandoth C., Schultz N., Cherniack A.D., Akbani R., Liu Y., Shen H., Robertson A.G., Pashtan I., Shen R., Cancer Genome Atlas Research Network (2013). Integrated Genomic Characterization of Endometrial Carcinoma. Nature.

[7] Nero C., Pasciuto T., Cappuccio S., Corrado G., Pelligra S., Zannoni G.F., Santoro A., Piermattei A., Minucci A., Lorusso D. (2022). Further Refining 2020 ESGO/ESTRO/ESP Molecular Risk Classes in Patients with Early-Stage Endometrial Cancer: A Propensity Score-Matched Analysis. Cancer.

[8] Leon-Castillo A., Horeweg N., Peters E.E.M., Rutten T., Ter Haar N., Smit V.T.H.B.M., Kroon C.D., Boennelycke M., Hogdall E., Hogdall C. (2022). Prognostic Relevance of the Molecular Classification in High-Grade Endometrial Cancer for Patients Staged by Lymphadenectomy and without Adjuvant Treatment. Gynecol. Oncol..

[9] Jamieson A., Vermij L., Kramer C.J.H., Jobsen J.J., Jürgemlienk-Schulz I., Lutgens L., Mens J.W., Haverkort M.A.D., Slot A., Nout R.A. (2023). Clinical Behavior and Molecular Landscape of Stage I P53-Abnormal Low-Grade Endometrioid Endometrial Carcinomas. Clin. Cancer Res..

[10] Bosse T., Nout R.A., McAlpine J.N., McConechy M.K., Britton H., Hussein Y.R., Gonzalez C., Ganesan R., Steele J.C., Harrison B.T. (2018). Molecular Classification of Grade 3 Endometrioid Endometrial Cancers Identifies Distinct Prognostic Subgroups. Am. J. Surg. Pathol..

[11] Casanova J., Babiciu A., Duarte G.S., da Costa A.G., Serra S.S., Costa T., Catarino A., Leitão M.M., Lima J. (2025). Abnormal P53 High-Grade Endometrioid Endometrial Cancer: A Systematic Review and Meta-Analysis. Cancers.

[12] Talhouk A., McConechy M.K., Leung S., Li-Chang H.H., Kwon J.S., Melnyk N., Yang W., Senz J., Boyd N., Karnezis A.N. (2015). A Clinically Applicable Molecular-Based Classification for Endometrial Cancers. Br. J. Cancer.

[13] Vermij L., Léon-Castillo A., Singh N., Powell M.E., Edmondson R.J., Genestie C., Khaw P., Pyman J., McLachlin C.M., Ghatage P. (2022). P53 Immunohistochemistry in Endometrial Cancer: Clinical and Molecular Correlates in the PORTEC-3 Trial. Mod. Pathol..

[14] Singh N., Piskorz A.M., Bosse T., Jimenez-Linan M., Rous B., Brenton J.D., Gilks C.B., Köbel M. (2020). P53 Immunohistochemistry Is an Accurate Surrogate for TP53 Mutational Analysis in Endometrial Carcinoma Biopsies. J. Pathol..

[15] Kommoss S., McConechy M.K., Kommoss F., Leung S., Bunz A., Magrill J., Britton H., Kommoss F., Grevenkamp F., Karnezis A. (2018). Final Validation of the ProMisE Molecular Classifier for Endometrial Carcinoma in a Large Population-Based Case Series. Ann. Oncol..

[16] Raffone A., Travaglino A., Mascolo M., Carotenuto C., Guida M., Mollo A., Insabato L., Zullo F. (2020). Histopathological Characterization of ProMisE Molecular Groups of Endometrial Cancer. Gynecol. Oncol..

[17] Talhouk A., McConechy M.K., Leung S., Yang W., Lum A., Senz J., Boyd N., Pike J., Anglesio M., Kwon J.S. (2017). Confirmation of ProMisE: A Simple, Genomics-Based Clinical Classifier for Endometrial Cancer. Cancer.

[18] Sakamoto I., Kagami K., Nozaki T., Hirotsu Y., Amemiya K., Oyama T., Omata M. (2023). P53 Immunohistochemical Staining and TP53 Gene Mutations in Endometrial Cancer: Does Null Pattern Correlate with Prognosis?. Am. J. Surg. Pathol..

[19] Köbel M., Ronnett B.M., Singh N., Soslow R.A., Gilks C.B., McCluggage W.G. (2019). Interpretation of P53 Immunohistochemistry in Endometrial Carcinomas: Toward Increased Reproducibility. Int. J. Gynecol. Pathol. Off. J. Int. Soc. Gynecol. Pathol..

[20] Rabban J.T., Garg K., Ladwig N.R., Zaloudek C.J., Devine W.P. (2021). Cytoplasmic Pattern P53 Immunoexpression in Pelvic and Endometrial Carcinomas with TP53 Mutation Involving Nuclear Localization Domains: An Uncommon But Potential Diagnostic Pitfall with Clinical Implications. Am. J. Surg. Pathol..

[21] Concin N., Matias-Guiu X., Vergote I., Cibula D., Mirza M.R., Marnitz S., Ledermann J., Bosse T., Chargari C., Fagotti A. (2021). ESGO/ESTRO/ESP Guidelines for the Management of Patients with Endometrial Carcinoma. Int. J. Gynecol. Cancer.

[22] Berek J.S., Matias-Guiu X., Creutzberg C., Fotopoulou C., Gaffney D., Kehoe S., Lindemann K., Mutch D., Concin N., Endometrial Cancer Staging Subcommittee (2023). FIGO Staging of Endometrial Cancer: 2023. Int. J. Gynecol. Obstet..

[23] Köbel M., Piskorz A.M., Lee S., Lui S., LePage C., Marass F., Rosenfeld N., Mes Masson A., Brenton J.D. (2016). Optimized P53 Immunohistochemistry Is an Accurate Predictor of TP53 Mutation in Ovarian Carcinoma. J. Pathol. Clin. Res..

[24] Heydt C., Fassunke J., Künstlinger H., Ihle M.A., König K., Heukamp L.C., Schildhaus H.-U., Odenthal M., Büttner R., Merkelbach-Bruse S. (2014). Comparison of Pre-Analytical FFPE Sample Preparation Methods and Their Impact on Massively Parallel Sequencing in Routine Diagnostics. PLoS ONE.

[25] Hoang L.N., Kinloch M.A., Leo J.M., Grondin K., Lee C.-H., Ewanowich C., Köbel M., Cheng A., Talhouk A., McConechy M. (2017). Interobserver Agreement in Endometrial Carcinoma Histotype Diagnosis Varies Depending on The Cancer Genome Atlas (TCGA)-Based Molecular Subgroup. Am. J. Surg. Pathol..

[26] Talhouk A., Hoang L.N., McConechy M.K., Nakonechny Q., Leo J., Cheng A., Leung S., Yang W., Lum A., Köbel M. (2016). Molecular Classification of Endometrial Carcinoma on Diagnostic Specimens Is Highly Concordant with Final Hysterectomy: Earlier Prognostic Information to Guide Treatment. Gynecol. Oncol..

[27] Stelloo E., Nout R.A., Naves L.C.L.M., ter Haar N.T., Creutzberg C.L., Smit V.T.H.B.M., Bosse T. (2014). High Concordance of Molecular Tumor Alterations between Pre-Operative Curettage and Hysterectomy Specimens in Patients with Endometrial Carcinoma. Gynecol. Oncol..

[28] Plotkin A., Kuzeljevic B., De Villa V., Thompson E.F., Gilks C.B., Clarke B.A., Köbel M., McAlpine J.N. (2020). Interlaboratory Concordance of ProMisE Molecular Classification of Endometrial Carcinoma Based on Endometrial Biopsy Specimens. Int. J. Gynecol. Pathol..

[29] Guo Q., Tang S., Ju X., Feng Z., Zhang Z., Peng D., Liu F., Du H., Wang J., Zhang Y. (2024). Identification of Molecular Subtypes for Endometrial Carcinoma Using a 46-Gene next-Generation Sequencing Panel: A Retrospective Study on a Consecutive Cohort. ESMO Open.

[30] Rios-Doria E., Momeni-Boroujeni A., Friedman C.F., Selenica P., Zhou Q., Wu M., Marra A., Leitao M.M., Iasonos A., Alektiar K.M. (2023). Integration of Clinical Sequencing and Immunohistochemistry for the Molecular Classification of Endometrial Carcinoma. Gynecol. Oncol..

