# Supplementary material for: Assessment of p53 in Endometrial Carcinoma Biopsy and Corresponding Hysterectomy Cases in a Real-World Setting: Which Cases Need Molecular Work-Up?
Source: Cancers (Basel). 2025 Apr 29;17(9):1506. doi: 10.3390/cancers17091506 (PMC12071035; doi:10.3390/cancers17091506)
Supplement: Supplementary file 1 [file cancers-17-01506-s001.zip › Supplementary Figure S1.docx]

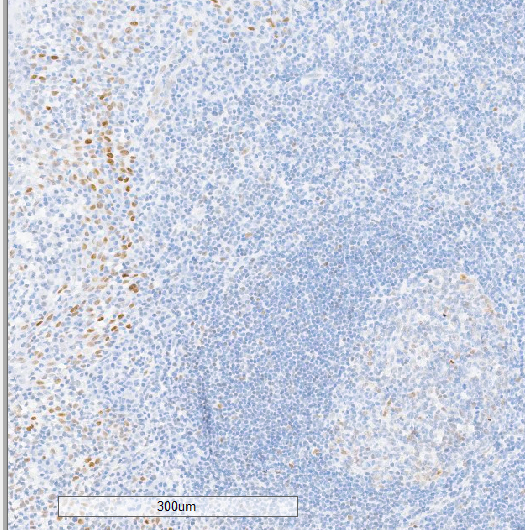

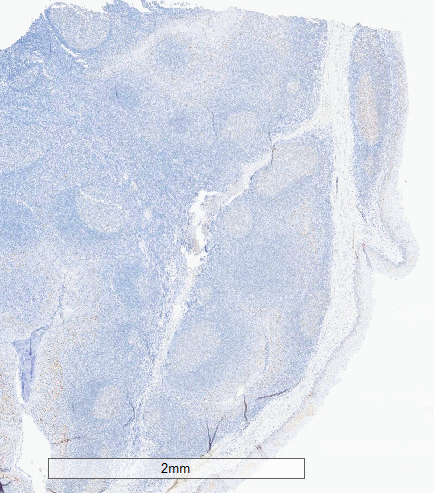


**B A**

**A A**


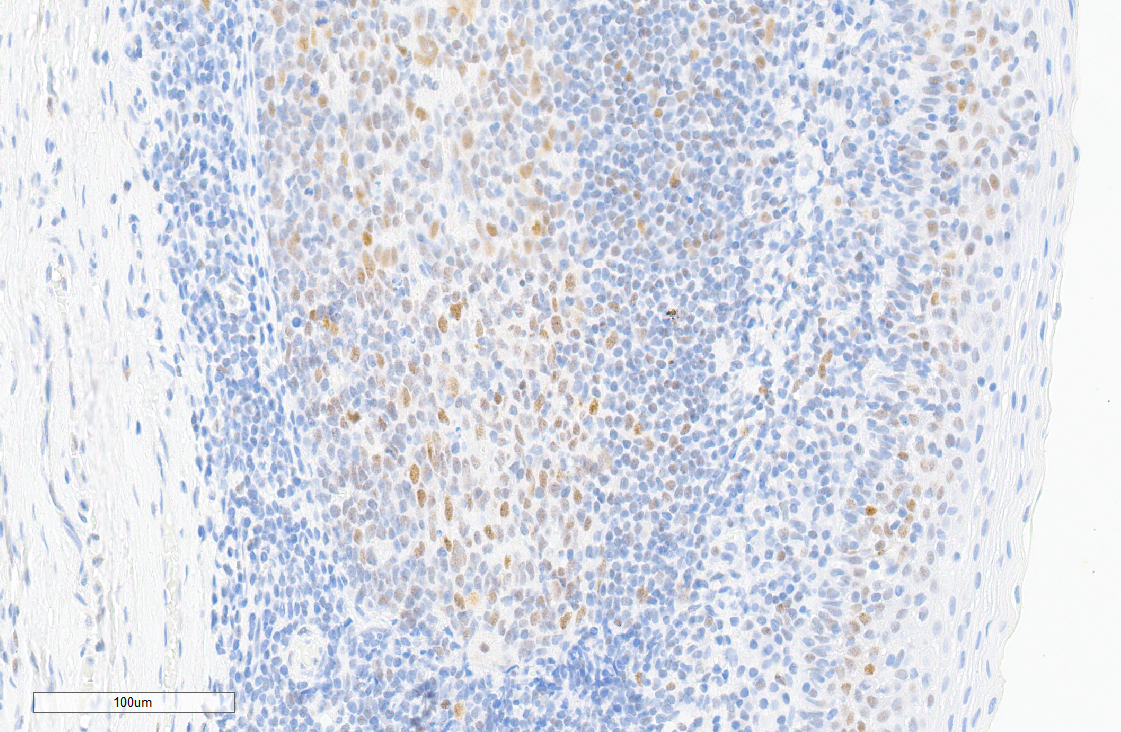


**C A**

**Supplementary Figure S1.** Negative control for p53 immunohistochemical staining

(A) Low magnification of tonsil tissue stained for p53. (B) High magnification of a germinal center in tosil tissue stained for p53. (C) High magnfication of tonsillar squamous epithelium stained for p53.
